# Supplementary material for: dGAMLSS: an exact, distributed algorithm to fit Generalized Additive Models for Location, Scale, and Shape for privacy-preserving population reference charts
Source: Bioinformatics. 2026 Jan 9;42(1):btaf625. doi: 10.1093/bioinformatics/btaf625 (PMC12802883; doi:10.1093/bioinformatics/btaf625)
Supplement: btaf625_Supplementary_Data [file btaf625_supplementary_data.zip › 5 Supplemental Materials.docx]

5 Supplemental Materials

5.1 Distributed smooth terms

We provide three distributed RS approaches of increasing complexity for fitting up to one smooth term per parameter. We first discuss using fixed effect smooth terms, where wiggliness of the smooth term is directly controlled by limiting the number of spline knots. Next, we describe fixed penalty smooth terms, where the spline basis is specified with more knots than necessary and wiggliness of the smooth term is controlled via a known penalty hyperparameter. Finally, we propose an algorithm for fully-automated penalty selection for smooth terms using either generalized Akaike Information Criterion (GAIC) or generalized cross-validation (GCV). For all smooth terms in dGAMLSS, we use B-splines, but other types of splines can also be used.

5.1.1 Distributed knot placement and smooth design matrix specification

To fit a given smooth term, dGAMLSS requires that a common spline basis be used across all $i$ sites. This basis must be chosen such that the global minimum and global maximum for the relevant covariate are contained within the boundary knots of the spline, an identical number of knots are used across sites, and knots are placed at identical locations across sites. Knots can either be placed at regular intervals over the range of the covariate values or at approximate quantiles of the relevant covariate for maximum efficiency. If fixed effect smooth terms are used, either knot placement approach can be used – regular interval knots are easier to place, while approximate quantiles are more efficient but require additional information to be sent in the first communication round. For fixed effect smooth terms, a total of $EDF-2$ knots should be placed, including the two boundary knots, in order to achieve a given desired EDF. If fixed penalty smooth terms are used, regular interval knot placement is adequate, given that enough knots are placed, and EDF is controlled using a penalty hyperparameter, discussed below.

One round of pre-fitting communication is required to specify the spline basis. If regular interval knots are desired, each site must send site-specific ranges for the relevant covariate such that the global range can be calculated. Knots can then be placed at regular intervals along this global range by the central site, and these knot locations can be sent back to other sites. If approximate quantile knots are desired, relevant summary statistics should also be simultaneously sent in this communication round, such as site sample size as well as site-specific covariate means and standard deviations (if the covariate distribution is thought to be nearly normal) or some number of quantiles (if the covariate distribution is thought to be non-normal). If site-specific covariate means and standard deviations are sent, these summary statistics and the site sample sizes can be used by the central site to estimate the pooled covariate mean and standard deviation and therefore obtain approximate quantiles. If site-specific quantiles, such as deciles are sent, the central site can simulate an appropriate number of uniformly distributed observations for each site within each decile, pool simulated observations across sites, and obtain approximate quantiles. These approximate quantiles can be sent back to other sites as the final knot locations. Once knots are placed, a common spline basis across all sites is achieved.

For fixed penalty or automated penalty smooth terms, additional information is necessary to learn the relationship between $\lambda_{k}$ and smooth term EDF, where each site must provide one additional round of pre-fitting communication containing site-specific matrices $\left[ \mathbf{X}_{ik} \mathbf{Z}_{ik} \right]^{T}\left[ \mathbf{X}_{ik} \mathbf{Z}_{ik} \right]$ for each parameter containing penalized smooths. To do so, a common spline basis is defined as above, and a joint penalty matrix across the fixed effects and smooth term is automatically specified based on the spline basis ([Eilers and Marx, 1996](#ref-eilersFlexibleSmoothingBsplines1996)). This joint penalty matrix for parameter $k$, $\mathbf{P}_{k}$, is a four-block square matrix of size $\text{length}\left( \boldsymbol{\beta}_{k} \right)+\text{length}\left( \boldsymbol{\gamma}_{k} \right)$, where the bottom right block matrix is the $\text{length}\left( \boldsymbol{\gamma}_{k} \right)\times\text{length}\left( \boldsymbol{\gamma}_{k} \right)$ spline penalty matrix and the other three block matrices are $\mathbf{0}$ matrices of appropriate size. This joint specification of the penalty matrix allows for direct fitting of the fixed effects and smooth terms without backfitting. Note that, if orthogonalization is performed for the smooth design matrix, the standard B-spline penalty matrix must be appropriately transformed using the $\mathbf{R}^{-1}$ matrix from above. Finally, the following formula is used to obtain the final EDF of the smooth term for any given penalty hyperparameter $\lambda_{k}$:

$$EDF=tr\left( \left( \sum_{i}^{m} \left[ \mathbf{X}_{ik} \mathbf{Z}_{ik} \right]^{T}\left[ \mathbf{X}_{ik} \mathbf{Z}_{ik} \right]+\lambda_{k}\mathbf{P}_{k} \right)^{-1}\left( \sum_{i}^{m} \left[ \mathbf{X}_{ik} \mathbf{Z}_{ik} \right]^{T}\left[ \mathbf{X}_{ik} \mathbf{Z}_{ik} \right]^{T} \right) \right)$$

Once necessary smooth design matrices and penalty matrices, as necessary, are defined, model fitting can proceed. Model fitting for fixed effect and fixed penalty models are described in Algorithm 1. Model fitting for automated penalty models is described in Algorithm 2. Notably, for automated penalty models, the default option of maximum likelihood penalty selection cannot be efficiently used in the distributed setting since the maximum likelihood algorithm requires iterative optimization of the penalty hyperparameter. Meanwhile, GAIC and GCV algorithms simply require selection of a penalty hyperparameter such that GAIC or GCV are minimized. In the distributed setting, testing GAIC or GCV across a grid of many hyperparameter values can be performed in one communication round.

5.2 Distributed multiple splines and interaction terms

Although multiple splines and interaction terms were not included in this analysis, they can be incorporated in dGAMLSS models. Multiple splines per parameter can be fit using fixed effect splines where each spline is limited in terms of degrees of freedom by the number of knots. Notably, for multiple spline models, including the spline-based interaction term models described below, pooled GAMLSS uses backfitting to avoid the problem of singular matrices, since each spline term incorporates an intercept term by design. Since, dGAMLSS circumvents backfitting for the sake of communication efficiency, appropriate columns must be removed from dGAMLSS spline-based interaction terms to avoid a singular design matrix. Even with such modification, instability in coefficients may result due to the multicollinearity of a near-singular matrix.

Interaction terms can be implemented via design matrix expansion prior to distributed estimation. Specifically, interactions between fixed effect covariates are handled by creating the appropriate indicator and product terms within each site’s design matrix. For example, an interaction between two categorical covariates can be encoded by replacing the original indicator columns with indicator variables for all pairwise combinations of categories. Similarly, categorical–continuous and continuous–continuous interactions can be represented by multiplying the relevant columns to generate additional covariates. After expansion, the resulting design matrix is passed to the distributed algorithm without modification to the estimation procedure. Inference on each expanded covariate is generated as above, while inference on the overall interaction term can be manually computed via the appropriate likelihood ratio test on the reduced model without interaction term.

Additionally, dGAMLSS can fit fixed effect categorical-continuous spline interactions via a similar approach where the spline basis is multiplied by the categorical variable indicators and one spline is fit for each level of the categorical variable. Fixed penalty categorical-continuous spline interactions can be fit using this same design matrix representation, but with a block diagonal penalty matrix, where each block on the diagonal is the penalty matrix for the corresponding category-specific spline, and off-diagonal blocks are the zero matrices of appropriate size. For fixed penalty interactions, a constant scalar penalty hyperparameter should be used for all category levels. Finally, fixed effect continuous-continuous spline interactions can be fit via replacement of the two sets of spline basis columns of the design matrix with pairwise multiplication of all spline basis terms. For spline-based interaction terms, a larger number of communication rounds may be required to fit complex multiple-spline models, and custom functions for likelihood ratio testing and plotting of final population reference charts are required.
